# Xanthine Oxidase Activity Is Associated with Risk Factors for Cardiovascular Disease and Inflammatory and Oxidative Status Markers in Metabolic Syndrome: Effects of a Single Exercise Session

**DOI:** 10.1155/2014/587083

**Published:** 2014-05-21

**Authors:** Ana Maria Pandolfo Feoli, Fabrício Edler Macagnan, Carla Haas Piovesan, Luiz Carlos Bodanese, Ionara Rodrigues Siqueira

**Affiliations:** ^1^Faculdade de Enfermagem, Nutrição e Fisioterapia, Pontifícia Universidade Católica do Rio Grande do Sul, Avenida Ipiranga 6681, Prédio 12, 8°andar, 90619-900 Porto Alegre, RS, Brazil; ^2^Programa de Pós-Graduação em Medicina e Ciências da Saúde, Pontifícia Universidade Católica do Rio Grande do Sul, Avenida Ipiranga 6690, 90610-000 Porto Alegre, RS, Brazil; ^3^Departamento de Farmacologia, Instituto de Ciências Básicas da Saúde, Universidade Federal do Rio Grande do Sul, Rua Sarmento Leite 500, 90050-170 Porto Alegre, RS, Brazil

## Abstract

*Objective*. The main goal of the present study was to investigate the xanthine oxidase (XO) activity in metabolic syndrome in subjects submitted to a single exercise session. We also investigated parameters of oxidative and inflammatory status. *Materials/Methods*. A case-control study (9 healthy and 8 MS volunteers) was performed to measure XO, superoxide dismutase (SOD), glutathione peroxidase activities, lipid peroxidation, high-sensitivity C-reactive protein (hsCRP) content, glucose levels, and lipid profile. Body mass indices, abdominal circumference, systolic and diastolic blood pressure, and TG levels were also determined. The exercise session consisted of 3 minutes of stretching, 3 minutes of warm-up, 30 minutes at a constant dynamic workload at a moderate intensity, and 3 minutes at a low speed. The blood samples were collected before and 15 minutes after the exercise session. *Results.* Serum XO activity was higher in MS group compared to control group. SOD activity was lower in MS subjects. XO activity was correlated with SOD, abdominal circumference, body mass indices, and hsCRP. The single exercise session reduced the SOD activity in the control group. *Conclusions*. Our data support the association between oxidative stress and risk factors for cardiovascular diseases and suggest XO is present in the pathogenesis of metabolic syndrome.

## 1. Introduction

The metabolic syndrome (MS) is mainly characterized by obesity, blood hypertension, hyperglycemia, or serum dyslipidemia as defined by the third report of the National Cholesterol Education Program Adult Treatment Panel III [1] and the International Diabetes Federation [2]. It has been demonstrated that the metabolic syndrome is associated with double risk of cardiovascular disease and mortality as well as stroke, even without type 2 diabetes mellitus [3]. However, the pathophysiological mechanisms by which the metabolic syndrome increases cardiovascular risk have not been fully clarified yet [4].

Epidemiological studies have also demonstrated the relationship between the uric acid and MS [5] and individual components of metabolic syndrome such as obesity, hypertension, dyslipidemia, [6], insulin resistance, higher C-reactive protein (CRP) concentration [7], and endothelial dysfunction [8].

According to Mankovsky et al. [9], cardiovascular events have also been related to uric acid levels. In addition, epidemiological studies demonstrated that uric acid is an independent risk factor for cardiovascular diseases [4, 10], particularly in hypertensive and diabetic individuals [11]. Although uric acid is considered an important water soluble antioxidant, several authors have discussed the dual role of uric acid as a detrimental or protective factor [12, 13]. Uric acid is generated by xanthine oxidase (XO); this enzyme catalyses the conversion of hypoxanthine to uric acid and superoxide anion radical [14]. Although hyperuricemia has been linked to metabolic syndrome, the role of xanthine oxidase remains poorly understood.

As described above, XO is a source of reactive oxygen species, generating superoxide anion radical. Several studies have reported that oxidative stress, the imbalance between free radical levels and antioxidant capacity, plays a significant role in the pathogenesis of cardiovascular disease [15] and diabetes [16]. However, few data are available concerning the associations between circulating concentrations of oxidative biomarkers and MS. Specifically, metabolic syndrome-related oxidative changes in macromolecules and antioxidant enzymatic system have been described, although these results are contradictory, pointing to a rather complex relationship to link oxidative status with the metabolic syndrome [4]. Besides, despite the oxidative status heterogeneity in different ethnic groups [17], the oxidative stress markers in metabolic syndrome in Brazilian patients have received little attention.

Furthermore, the inflammation process has been widely connected to metabolic syndrome. Adipose tissue produces proinflammatory cytokines, called adipocytokines, which could increase C-reactive protein (CRP) synthesis [16]. The ultrasensitive C-reactive protein (hsCRP) has been widely used to evaluate vascular inflammation and cardiovascular risk [18]. Interestingly, cytokines can irreversibly convert endothelial xanthine dehydrogenase to its active form, XO [19].

Epidemiological and experimental studies have demonstrated that exercise may be able to prevent and treat metabolic syndrome and its components, such as type 2 diabetes and cardiovascular disease [20]. The beneficial effect has been related to training-induced adaptations in oxidative status; this hypothesis is based on the fact that acute exercise session would induce an increase in reactive species levels, although to our knowledge there are no studies reporting the impact of acute exercise on oxidative status in metabolic syndrome.

Our working hypothesis was that xanthine oxidase activity is increased in metabolic syndrome and this parameter is correlated to clinical criteria, oxidative stress, and inflammatory markers and the acute exercise would be able to alter these parameters.

The main goal of the present study was to investigate the plasma XO activity in metabolic syndrome patients. We also investigated lipid peroxidation and antioxidant enzyme activities, namely, superoxide dismutase and glutathione peroxidase. Besides, CRP levels were detected as a low-grade inflammatory marker. The acute effects of single exercise session on these parameters were also investigated.

## 2. Methods

### 2.1. Subjects

Subjects were examined by the same physician at the Rehabilitation Center, Pontifical Catholic University of Rio Grande do Sul state (PUCRS). The characteristics of the subjects are shown in Table 1. The study included 17 subjects (09 men and 08 women; mean age: 50.28 ± 6.5 years). The control group consisted of 9 healthy subjects, and 8 patients were diagnosed with MS. All subjects were sedentary with no regular physical activity. Based on previous report comparing plasma XO activity between control subjects and patients with hyperlipidemia [21], a sample size of 7 subjects in each group was needed to detect the effect for a 90% power at a 0.05 significance level.

The diagnostic criteria for MS were made using the NCEP ATP III [1], following three or more criteria [11]: abdominal obesity based on abdominal circumference (men, >102 cm; women, >88 cm); triglycerides (≥150 mg/dL); high-density lipoprotein- (HDL-) cholesterol (men, <40 mg/dL; women, <50 mg/dL); blood pressure (≥130/85 mm Hg); and fasting glucose (≥110 mg/dL). Exclusion criteria were clinical manifestations of cardiovascular disease, diabetes, hypertension, other chronic diseases, renal or hepatic insufficiency and hypothyroidism, use of drugs capable of modifying the lipid profile, inflammation that could not be withdrawn 6 weeks before initiating the study, and any infection or inflammatory disease within the 6 weeks prior to the study. The study protocol and the procedures were approved by Ethics Committee of PUCRS (0603024) and the subjects gave informed consent.

### 2.2. Chemicals

All chemicals used in this study were purchased from Sigma Chemical Co. (St. Louis, MO, USA) and were of analytical grade or the highest grade available.

### 2.3. Clinical and Anthropometric Parameters

Blood pressure was measured with subjects in the sitting position using a mercury sphygmomanometer after a 10-min period of rest, two separated measurements being performed. Anthropometrical measurements included weight, height, and abdominal circumference. Weight was obtained using calibrated scales (Filizola, Brazil) while subjects wore light clothing and no shoes and height was measured by a fixed stadiometer. BMI was calculated as weight divided by height square. Abdominal circumference was measured in orthostatic position at the midpoint between the low costal rim and the iliac crest.

### 2.4. Physical Activity Session

The physical activity session was supervised at all times and took about 40 minutes divided as follows: 3 minutes spent in stretching, 3 minutes in warming up (walking on the treadmill at 3.2 km/h without grate), 30 minutes in a constant dynamic workload on moderate intensity (speed and grate were sufficiently increased to reach the set point of the heart rate (HR), and 3 minutes at a low velocity (3.2 km/h) to cool down. A HR monitor (POLAR) was used to control and keep the HR on target as suggested by the Brazilian Guideline for MS diagnosis and treatment [11]. The range of the heart rate (HR) between 65 and 75% of the age predicted maximum HR is a common method. This method was used to set individual workload on treadmill (Inbrasport, Export model). The velocity and grate of the treadmill were progressively increased until HR reached the set point.

### 2.5. Biochemical Parameters

All blood samples were collected in the morning after overnight fast (before exercise) and 15 minutes after the acute exercise session. Blood samples were collected in heparinized tubes and immediately centrifuged at room temperature for 10 min at 3000 rpm. The supernatant was transferred to cryotubes and aliquots were stored at −70°C until assay for xanthine oxidase (XO), superoxide dismutase (SOD), glutathione peroxidase (GSHPx), lipid peroxidation (TBARS), high-sensitivity CRP, glucose, and lipid parameters were determined. Plasma glucose, total serum cholesterol, serum triglyceride, and serum HDL-cholesterol levels were measured by standard enzymatic methods using reagents in a fully automated analyzer (Vitros 950 dry chemistry system; Johnson & Johnson, Rochester, NY). LDL-cholesterol was estimated using the Friedewald equation [22]. High-sensitivity CRP (hsCRP) was measured using the ADVIA Centaur immunoassay on the ADVIA Centaur analyzer (Siemens Medical Solutions Diagnostics, Frimley, Surrey, UK).

### 2.6. Atherogenic Index of Plasma (AIP)

The new atherogenic plasma index (AIP) is a logarithmic transformation of the ratio of the molar triglyceride (TG) concentration and high density lipoprotein cholesterol (HDL-C). AIP correlates closely with the particle size of LDL (*r* = 0.8) and the esterification rate of plasma cholesterol devoid of apo B lipoproteins (FERHDL), *r* = 0.9, which are considered at present the most sensitive indicators of the atherogenic plasma profile. The mean AIP values of nonrisk groups equaled zero or were lower, while atherogenic risk AIP reached positive values [7].

### 2.7. Determination of XO Activity

Serum XO activity (cytoplasmic xanthine oxidase (EC1.17.3.2)) was measured according to the method of Prajda and Weber, where activity is measured by determination of uric acid from xanthine [23]. Serum was incubated for 30 min at 37°C in phosphate buffer (pH 7.5, 50 mM) containing xanthine (4 mM). The reaction was stopped adding 20 *μ*L 100% TCA. The mixture was then centrifuged at 4000 ×g for 20 min. Uric acid was determined in the supernatant by absorbance at 292 nm against a blank. The results are expressed as units per milliliter (U/mL).

### 2.8. Determination of SOD Activity

Superoxide dismutase activity was determined with a RANSOD kit (Randox Laboratories, San Diego, CA, USA). This method employs xanthine and xanthine oxidase to generate superoxide radical, which reacts with 2-(4-iodophenyl)-3-(4-nitrophenol)-5-phenyltetrazolium chloride to form a red formazan dye that is assayed spectrophotometrically at 505 nm and 37°C. Inhibition of production of the chromogen is proportional to SOD activity in the sample. SOD activity was expressed as units per *μ*g protein.

### 2.9. Determination of GPx Activity

GPx activity was determined according to Wendel [24]. The reaction was carried out at 25°C in 600 *μ*L of solution containing 100 mM pH 7.7 potassium phosphate buffer, 1 mM EDTA, 0.4 mM sodium azide, 2 mM GSH, 0.1 mM NADPH, and 0.62 U of GSH reductase. The activity of selenium-dependent GPx was measured taking* tert*-butyl hydroperoxide as substrate at 340 nm. The contribution of spontaneous NADPH oxidation was always subtracted from the overall reaction rate. GPx activity was expressed as nmol NADPH oxidized per minute per mg protein.

### 2.10. Lipid Peroxidation (TBARS)

The formation of thiobarbituric acid reactive substances (TBARS) was based on the methods described by Buege [25]. Aliquots of samples were incubated with 10% trichloroacetic acid (TCA) and 0.67% thiobarbituric acid (TBA). The mixture was heated in a boiling water bath. Afterwards,* n*-butanol was added and the mixture was centrifuged. The organic phase was collected to measure fluorescence at excitation and emission wavelengths of 515 and 553 nm [26], respectively; 1,1,3,3-tetramethoxypropane, which is converted to malondialdehyde (MDA), was used as standard. The results were expressed as nmol MDA formed/mg protein.

### 2.11. Statistical Analysis

Statistical evaluation was carried out with the SPSS 11.0 (Statistical Packages for Social Sciences; SPSS Inc, Chicago, Illinois, USA). Results were expressed as mean (±SD). Baseline results were compared by the Student's *t*-test (see Table 1). Pearson correlation and linear regression analysis were used to study the relationships between all evaluated parameters. To test the effect of single session treadmill on oxidative status markers in patients with metabolic syndrome, we used two-way ANOVA for repeated measurements. The post hoc test was performed by Tukey test. A value of *P* ≤ 0.05 was considered significant.

## 3. Results

Baseline clinical and biochemical characteristics of control and MS groups are shown in Table 1. MS subjects showed higher levels of BMI, abdominal circumference, systolic and diastolic blood pressure, and TG levels, while HDL-C was decreased in this group.

**Figure 1:**
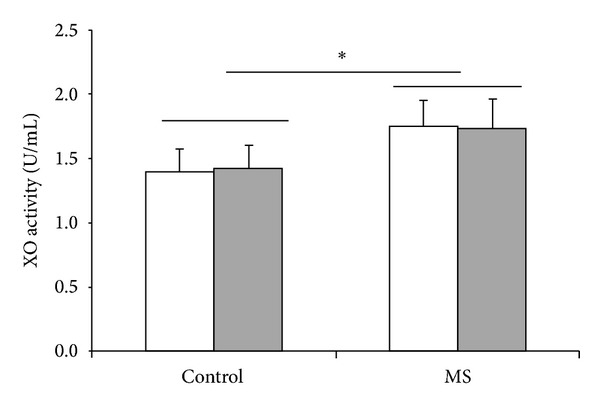
Effects of metabolic syndrome (MS) and single treadmill session on XO activity. White bar = before exercise; shadowed bar = after exercise; two-way ANOVA followed by Tukey test. *Significant difference between control and SM groups; *P* < 0.05.

**Figure 2:**
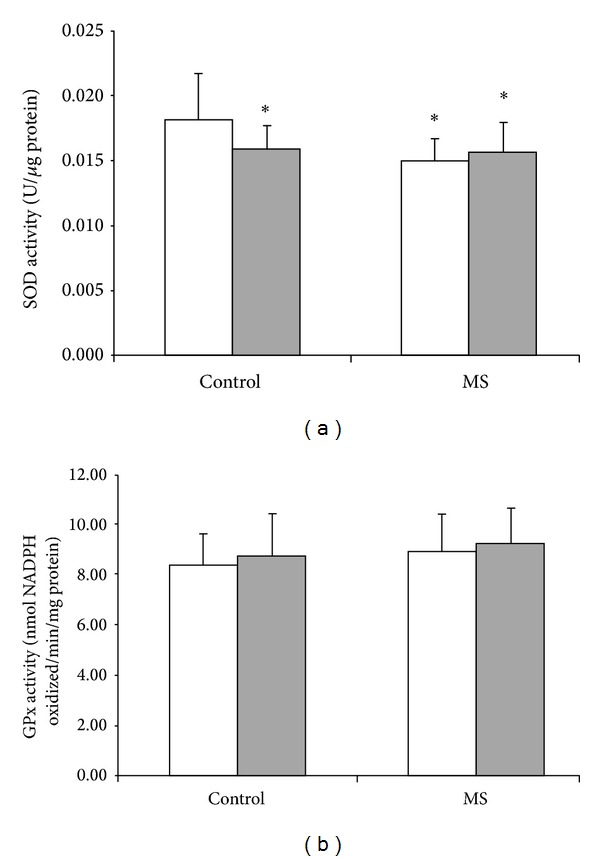
Effects of metabolic syndrome (MS) and single treadmill session on antioxidant enzymes activities, SOD (a) and GPx (b). White bar = before exercise; shadowed bar = after exercise; two-way ANOVA followed by Tukey test. *Significantly different as compared to control group before acute exercise; *P* < 0.05.

**Figure 3:**
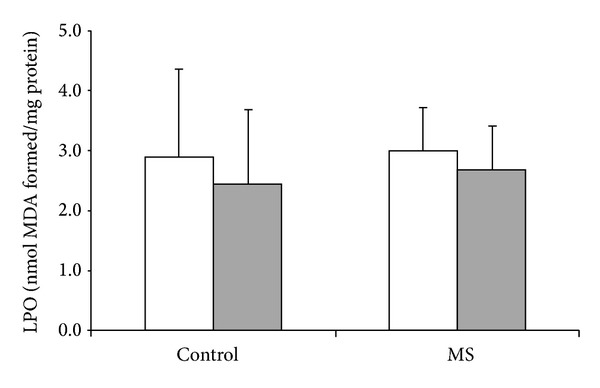
Effects of metabolic syndrome (MS) and single treadmill session on lipid peroxidation through TBARS. White bar = before exercise; shadowed bar = after exercise; two-way ANOVA followed by Tukey test.

MS was able to alter some oxidative stress parameters, especially XO activity which increased in MS group (*P* = 0.002, Figure 1). The impact of MS and acute exercise on the antioxidant enzymes studied is presented in Figure 2. SOD activity was reduced in MS group (Figure 2(a); *P* = 0.019). On the other hand, no differences on the GSHPx activity were found in MS subjects (Figure 2(b)).  Figure 3 illustrates the effect of MS and acute exercise on lipid peroxidation evaluated by TBARS levels. MS did not alter significantly TBARS levels.

There was a significant correlation between XO activity and metabolic syndrome markers. Pearson correlation coefficients between XO activity and anthropometric and biochemical and oxidative stress parameters are shown in Table 2. XO activity was correlated positively with BMI, abdominal waist, and hsCRP and negatively with SOD activity. Besides, there was a significant correlation between SOD activity and metabolic syndrome markers (data not shown).

The single exercise session induced a reduction in SOD activity (Figure 2(a)) in the control group, without any effect in MS group. This exercise protocol did not alter XO activity, TBARS levels, and GSHPx activity.

## 4. Discussion

This study adds evidence to the pathophysiological mechanisms in the metabolic syndrome, principally to role of oxidative stress in MS and its components.

Our results provide the first evidence that higher XO activity may have a central role in MS. MS subjects showed increased XO activity; besides XO activity was correlated to metabolic syndrome markers. Accordingly, uric acid levels have been related to cardiovascular events [9], which is relevant since XO is a metabolic pathway for uric acid generation. It has been suggested that higher uric acid levels are associated with their lower renal excretion [27], although the results presented here could suggest an increase in uric acid production induced by augmented XO activity. Nevertheless, our results do not exclude the participation of lower renal excretion.

Also, it is important to note that uric acid nephrolithiasis is significantly more frequent among patients with the metabolic syndrome and obesity [28], which can be related to enhanced xanthine oxidase activity. Moreover, it has been demonstrated that there is an association between kidney stones and atherosclerosis [29].

It is important to note that excessive dietary sucrose or high-fructose corn syrup has been related to risk factors for cardiovascular disease and metabolic syndrome. It has been suggested that this effect is linked to increased uric acid levels produced by fructokinase pathway [30]. Then a causal role of uric acid in fructose-induced MS has been hypothesized; however it can be supposed that altered XO activity and fructose-induced higher levels of uric acid can produce synergistic deleterious effect.

In addition, our results could provide a new perspective for pharmacological prevention and/or MS treatment, at least as adjuvant strategies. In this sense, XO inhibitors, such as allopurinol and oxypurinol, have been suggested for the prevention of cardiovascular diseases [14].

Considering that an increase in XO activity was observed, whose reactions have superoxide anion radical as a byproduct [31], the role of this enzyme in MS might be suggested in producing an imbalance between free radical levels and antioxidant capacity. Our results corroborate the hypothesis that oxidative stress is involved in MS pathogenesis.

Additionally, in an effort to compare our findings with results reported in the literature, we also investigated some parameters of oxidative status, lipid peroxidation, and antioxidant enzyme activities, namely, superoxide dismutase and glutathione peroxidase.

SOD activity, a major intracellular and extracellular enzymatic defense system against superoxide, was significantly lower in MS subjects, and there was a negative correlation between SOD and XO activities. An increased XO activity generating superoxide anion radical and a concomitantly decreased SOD activity were found in MS patients of this study, indicating an increase in production and a decrease in removal of superoxide radicals. This disturbed superoxide content may be important in the pathogenesis of MS, since excessive content of reactive species may induce endothelial dysfunction [16, 32], which is associated with different stages of atherosclerosis [33–35]. Moreover, in agreement with Isogawa et al. [21], SOD activity was negatively correlated with some metabolic syndrome markers, namely, abdominal waist, BMI, and hsCRP.

It is important to note that SOD activity results are contradictory in different ethnicities, since Japanese and Taiwanese MS patients have significantly lower SOD activity [36, 37], while Caucasians recruited in Prague demonstrated higher SOD activity [6]. These findings support the idea that oxidative parameters must be tested in specific populations [17].

The evaluated metabolic syndrome parameters can be related to endothelial cell dysfunction. In this context, our findings can corroborate the hypothesis that endothelial dysfunction is related to decreased nitric oxide (NO) content through inactivation of NO by superoxide, considering that NO modulates vascular tone through its vasodilator action [38]. The interaction of NO with superoxide may yield peroxynitrite, which breaks down to form a hydroxyl radical, thereby resulting in increased oxidative stress [39].

In contrast to previous data, MS did not modify TBARS levels. It is possible to infer that TBARS levels were unchanged because glutathione peroxidase activity, which breaks down peroxides (notably those derived from the oxidation of membrane phospholipids), remained unaltered [37]. This finding may suggest that increases in lipoperoxidation levels may not be crucial in MS; therefore, other molecular mechanisms cannot be ruled out.

This work demonstrated that XO activity correlated positively with hsCRP, a marker of low-grade inflammation. Also, Martinez-Hervas et al. [40] found a positive association between XO activity and hsCRP in a study with familial combined hyperlipidemia. It is possible to infer that inflammatory mediators, such as cytokines, are involved in the activation of XO [19]. It is interesting to comment that a growing body of evidence suggests that inflammation markers are closely related to MS and its consequences [33].

Our data indicate an association between increased abdominal circumference and XO activity. It is possible to hypothesize that XO-generated superoxide can be, at least in part, related to visceral adiposity. Obesity, characterized by enlarged adipocytes, and insulin resistance are associated with impaired adipogenesis and a low-grade chronic inflammation. Visceral adiposity plays an important role in the progression of cardiovascular diseases, insulin resistance, and type 2 diabetes [32, 34].

Although regular exercise has been suggested for the management of MS and its components, changes in human performance and physiological conditions after acute exercise session have only recently attracted attention. It is important to describe that the beneficial effect of exercise has been linked to free radicals production achieving adaptations in antioxidant system. Considering this hypothesis, we could expect that a single session of exercise would be able to induce an oxidative stress status; however, our acute session exercise protocol reduced SOD activity in healthy control subjects, without any effect in MS group, showing different profiles between these groups. It is impossible to establish, at this moment, a mechanism by which the exercise alters this parameter only in healthy subjects, although it is possible to postulate that MS condition may induce sustained adaptations which a single exercise session was unable to impact. Besides, the physiological impact of this effect in healthy subjects remains unsolved, since reduced SOD activity could increase the superoxide radical content, although it might reduce the hydrogen peroxide generation, as reactive oxygen species that combined to metal iron would originate hydroxyl radicals, inducing a hazardous state.

It is possible to suggest that types, regimen, and intensities of physical activity protocols can be determinant to impact oxidative status parameters. In this context, it is interesting to note that walking and stretching or resistance exercise training during 12 weeks without diet-induced weight loss did not alter oxidative stress in middle-aged men with MS [41]. Besides, aerobic exercise or Hatha yoga for 12 weeks did not alter lipid or protein oxidative damage [42], while longer exercise protocols have attenuated oxidative stress parameters in patients with type 2 diabetes [42, 43].

In accordance with the results presented here, the acute session exercise did not alter the plasma levels of pro- or anti-inflammatory cytokines in women with MS [44]; however, Greene et al. demonstrated that acute exercise increased hsCRP in subjects [45].

Our results support the hypothesis that increased XO activity has a central role in metabolic syndrome. The single exercise session protocol was able to alter SOD activity in healthy control group, without any impact on the MS subjects. Additional work will be required to investigate the molecular mechanisms behind these findings.

## Figures and Tables

**Table 1:** Clinical characteristics of the study populations.

Parameters	Controls (n = 9)	MS (n = 8)	P
Sex (F/M)	(5/4)	(4/4)	
Age (years)	50.56 ± 5.36	50.00 ± 7.69	0.863
BMI (kg/m^2^)	24.47 ± 1.10	37.14 ± 5.19	<0.001
AC (cm)			
Female	74.60 ± 9.69	114.63 ± 10.96	0.001
Male	94.00 ± 4.69	120.88 ± 10.38	0.003
Total-C (mg/dL)	198.44 ± 31.16	218.88 ± 39.77	0.253
LDL-C (mg/dL)	167.60 ± 29.28	167.30 ± 28.82	0.983
HDL-C (mg/dL)			
Female	63.80 ± 10.13	48.50 ± 7.59	0.041
Male	63.50 ± 4.95	43.67 ± 8.02	0.048
TG (mg/dL)	90.67 ± 30.81	211.25 ± 76.30	<0.001
AIP	0.13 ± 0.18	0.63 ± 0.21	<0.001
Glucose (mmol/L)	86.00 ± 5.32	104.38 ± 13.50	0.001
Insulin	4.23 ± 1.52	19.84 ± 5.80	0.003
HOMA	0.90 ± 0.34	5.22 ± 2.12	0.000
hsCRP (mg/dL)	0.12 ± 0.09	0.79 ± 0.29	<0.001
SBP (mmHg)	115.56 ± 13.79	145.13 ± 10.97	0.002
DBP (mmHg)	77.11 ± 13.38	93.75 ± 8.76	0.009

Data are expressed as mean ± SD. BMI: body mass indices, AC: abdominal circumference, Total-C: total cholesterol, LDL-C: low density lipoprotein cholesterol, HDL-C: high density lipoprotein cholesterol, TG: triglycerides, AIP: log.(TG/HDL-c); atherogenic index of plasma, HOMA: ((Glucose/18) ∗ insulin)/22,5; homeostatic model assessment for insulin resistance, hsCPR: high sensitive C protein reactivity, SBP: systolic blood pressure, DBP: diastolic blood pressure. Student's *t*-test.

**Table 2:** Pearson correlation with XO and clinical parameters (*n* = 17).

XO activity versus	*r*	*P*
SOD activity	−0.71	0.005
Abdominal circumference	0.61	0.010
BMI	0.66	0.007
hsCRP	0.70	0.005

BMI: body mass indices; hsCRP: high sensitive reactive C protein. Pearson correlation and linear regression analysis were used to study the relationships between parameters.

## Abbreviations

MS: Metabolic syndrome
NCEP ATP III: Third Report of the National Cholesterol Education Program Adult Treatment Panel III
XO: Xanthine oxidase
SOD: Superoxide dismutase
GSHPx: Glutathione peroxidase
hsCRP: High-sensitivity CRP
TBARS: Thiobarbituric acid reactive substances
NO: Nitric oxide.

## References

[1] National Institutes of Health (2001). *ATP III Guidelines At-A-Glance Quick Desk Reference*.

[2] Atlas Diabetes International Diabetes Federation. http://www.idf.org/diabetesatlas.

[3] Mottillo S, Filion KB, Genest J (2010). The metabolic syndrome and cardiovascular risk: a systematic review and meta-analysis. *Journal of the American College of Cardiology*.

[4] Alberti KGMM, Eckel RH, Grundy SM (2009). Harmonizing the metabolic syndrome: a joint interim statement of the international diabetes federation task force on epidemiology and prevention; National heart, lung, and blood institute; American heart association; World heart federation; International atherosclerosis society; And international association for the study of obesity. *Circulation*.

[5] Sui X, Church TS, Meriwether RA, Lobelo F, Blair SN (2008). Uric acid and the development of metabolic syndrome in women and men. *Metabolism: Clinical and Experimental*.

[6] Vávrová L, Kodydková J, Zeman M (2013). Altered activities of antioxidant enzymes in patients with metabolic syndrome. *Obesity Facts*.

[7] Dobiasova M (2006). AIP-atherogenic index of plasma as a significant predictor of cardiovascular risk: from research to practice. *Vnitřní lékařnství*.

[8] Farquharson CAJ, Butler R, Hill A, Belch JJF, Struthers AD (2002). Allopurinol improves endothelial dysfunction in chronic heart failure. *Circulation*.

[9] Mankovsky B, Kurashvili R, Sadikot S (2010). Is serum uric acid a risk factor for atherosclerotic cardiovascular disease?: a review of the clinical evidence. Part 1. *Diabetes and Metabolic Syndrome: Clinical Research and Reviews*.

[10] Kanbay M, Segal M, Afsar B, Kang DH, Rodriguez-Iturbe B, Johnson RJ (2013). The role of uric acid in the pathogenesis of human cardiovascular disease. *Heart*.

[11] Brandão A, Brandão AA, da Rocha Nogueira A (2005). I Diretriz brasileira de diagnóstico e tratamento da síndrome metabólica. *Brasileiros de Cardiologia*.

[12] Ioachimescu AG, Brennan DM, Hoar BM, Hazen SL, Hoogwerf BJ (2008). Serum uric acid is an independent predictor of all-cause mortality in patients at high risk of cardiovascular disease: a Preventive Cardiology Information System (PreCIS) database cohort study. *Arthritis and Rheumatism*.

[13] Okamoto K, Eger BT, Nishino T, Kondo S, Pai EF, Nishino T (2003). An extremely potent inhibitor of xanthine oxidoreductase: crystal structure of the enzyme-inhibitor complex and mechanism of inhibition. *The Journal of Biological Chemistry*.

[14] Puddu P, Puddu GM, Cravero E, Vizioli L, Muscari A (2012). The relationships among hyperuricemia, endothelial dysfunction, and cardiovascular diseases: molecular mechanisms and clinical implications. *Journal of Cardiology*.

[15] Furukawa S, Fujita T, Shimabukuro M (2004). Increased oxidative stress in obesity and its impact on metabolic syndrome. *Journal of Clinical Investigation*.

[16] Palmieri VO, Grattagliano I, Portincasa P, Palasciano G (2006). Systemic oxidative alterations are associated with visceral adiposity and liver steatosis in patients with metabolic syndrome. *The Journal of Nutrition*.

[17] Galassetti P (2012). Inflammation and oxidative stress in obesity, metabolic syndrome, and diabetes. *Experimental Diabetes Research*.

[18] Devaraj S, Singh U, Jialal I (2009). Human C-reactive protein and the metabolic syndrome. *Current Opinion in Lipidology*.

[19] Vorbach C, Harrison R, Capecchi MR (2003). Xanthine oxidoreductase is central to the evolution and function of the innate immune system. *Trends in Immunology*.

[20] Valko M, Leibfritz D, Moncol J, Cronin MTD, Mazur M, Telser J (2007). Free radicals and antioxidants in normal physiological functions and human disease. *International Journal of Biochemistry and Cell Biology*.

[21] Isogawa A, Yamakado M, Yano M, Shiba T (2009). Serum superoxide dismutase activity correlates with the components of metabolic syndrome or carotid artery intima-media thickness. *Diabetes Research and Clinical Practice*.

[22] Friedewald WT, Levy RI, Fredrickson DS (1972). Estimation of the concentration of low-density lipoprotein cholesterol in plasma, without use of the preparative ultracentrifuge. *Clinical Chemistry*.

[23] Prajda N, Weber G (1975). Malignant transformation linked imbalance: decreased xanthine oxidase activity in hepatomas. *FEBS Letters*.

[24] Wendel A, Jakoby WB (1980). Glutathione peroxidase. *Enzymatic Basis of Detoxification*.

[25] Buege J, Fleischer S, Packer L (1978). Microsomal lipid peroxidation. *Methods in Enzymology*.

[26] Yagi K (1998). Simple assay for the level of total lipid peroxides in serum or plasma. *Methods in Molecular Biology*.

[27] Facchini F, Chen Y-DI, Hollenbeck CB, Reaven GM (1991). Relationship between resistance to insulin-mediated glucose uptake, urinary uric acid clearance, and plasma uric acid concentration. *Journal of the American Medical Association*.

[28] Pasalic D, Marinkovic N, Feher-Turkovic L (2012). Uric acid as one of the important factors in multifactorial disorders—facts and controversies. *Biochemia Medica*.

[29] Fujii Y, Okada A, Yasui T (2013). Effect of adiponectin on kidney crystal formation in metabolic syndrome model mice via inhibition of inflammation and apoptosis. *PloS ONE*.

[30] Stanhope K, Schwarz J, Havel P (2013). Adverse metabolic effects of dietary fructose: results from the recent epidemiological, clinical, and mechanistic studies. *Current Opinion in Lipidology*.

[31] Baskol G, Baskol M, Kocer D (2007). Oxidative stress and antioxidant defenses in serum of patients with non-alcoholic steatohepatitis. *Clinical Biochemistry*.

[32] Kin HL, Yu LC, Wing BC, Chan JCN, Chu CWW (2006). Mesenteric fat thickness is an independent determinant of metabolic syndrome and identifies subjects with increased carotid intima-media thickness. *Diabetes Care*.

[33] Gustafson B, Hammarstedt A, Andersson CX, Smith U (2007). Inflamed adipose tissue: a culprit underlying the metabolic syndrome and atherosclerosis. *Arteriosclerosis, Thrombosis, and Vascular Biology*.

[34] Gustafson B (2010). Adipose tissue, inflammation and atherosclerosis. *Journal of Atherosclerosis and Thrombosis*.

[35] Villanova N, Moscatiello S, Ramilli S (2005). Endothelial dysfunction and cardiovascular risk profile in nonalcoholic fatty liver disease. *Hepatology*.

[36] Yokota T, Kinugawa S, Yamato M (2013). Systemic oxidative stress is associated with lower aerobic capacity and impaired skeletal muscle energy metabolism in patients with metabolic syndrome. *Diabetes Care*.

[37] Chen S, Yen CH, Huang YC, Lee BJ, Hsia S, Lin PT (2012). Relationships between inflammation, adiponectin, and oxidative stress in metabolic syndrome. *PLoS ONE*.

[38] Huang PL (2009). eNOS, metabolic syndrome and cardiovascular disease. *Trends in Endocrinology and Metabolism*.

[39] Jourd’heuil D, Jourd’heuil FL, Kutchukian PS, Musah RA, Wink DA, Grisham MB (2001). Reaction of superoxide and nitric oxide with peroxynitrite. Implications for peroxynitrite-mediated oxidation reactions in vivo. *The Journal of Biological Chemistry*.

[40] Martinez-Hervas S, Real JT, Ivorra C (2010). Increased plasma xanthine oxidase activity is related to nuclear factor kappa beta activation and inflammatory markers in familial combined hyperlipidemia. *Nutrition, Metabolism and Cardiovascular Diseases*.

[41] Venojärvi M, Korkmaz A, Wasenius N (2013). 12 Weeks' aerobic and resistance training without dietary intervention did not influence oxidative stress but aerobic training decreased atherogenic index in middle-aged men with impaired glucose regulation. *Food and Chemical Toxicology*.

[42] Gordon LA, Morrison EY, McGrowder DA (2008). Effect of exercise therapy on lipid profile and oxidative stress indicators in patients with type 2 diabetes. *BMC Complementary and Alternative Medicine*.

[43] Lazarevic G, Antic S, Cvetkovic T, Djordjevic V, Vlahovic P, Stefanovic V (2008). Effects of regular exercise on cardiovascular risk factors profile and oxidative stress in obese type 2 diabetic patients in regard to SCORE risk. *Acta Cardiologica*.

[44] Pereira G, Tibana RA, Navalta J (2012). Acute effects of resistance training on cytokines and osteoprotegerin in women with metabolic syndrome. *Clinical Physiology and Functional Imaging*.

[45] Greene N, Martin S, Crouse S (2012). Acute exercise and training alter blood lipid and lipoprotein profiles differently in overweight and obese men and women. *Obesity*.

